# Where to Plant Trees? Designing Net-Zero Industrial Landscapes that Promote Public Health

**DOI:** 10.1021/acs.est.6c00851

**Published:** 2026-04-23

**Authors:** Michael Charles, Bhavik R. Bakshi

**Affiliations:** † Department of Biological and Environmental Engineering, 5922 Cornell University, Ithaca, New York 14853, United States; ‡ William G. Lowrie Department of Chemical and Biomolecular Engineering, The Ohio State University, Columbus, Ohio 43210, United States; § School for Engineering of Matter, Transport and Energy, School of Sustainability, School of Complex Adaptive Systems, 7864 Arizona State University, Tempe, Arizona 85281, United States

**Keywords:** sustainable design, ecosystem services, carbon neutrality, nature-based solutions, air quality regulation, techno-ecological synergy

## Abstract

To contribute to the development of holistic sustainability strategies, we present a computational sustainable design framework for techno-ecological systems that integrates ecosystem coservices into the pursuit of carbon neutrality and net-positive industries. Public-health impacts of land-based pollution mitigation strategies are explicitly modeled and implemented within an integrated optimization framework, enabling the simultaneous evaluation of climate mitigation and local health outcomes. This nature-positive and people-positive approach identifies where land-use changes (LUCs) can most effectively reduce pollutant exposure and health-related incidences, providing practical guidance for prioritizing locations for nature-based solutions. A power generation case study is presented to compare this health-based social benefit approach with conventional and prior techno-ecological approaches. Highlighting LUC spatial importance, our study shows that 97% of averted mortalities may be lost between best- and worst-case scenarios. Our results suggest that cost-benefit analyses of land-use changes focused solely on carbon uptake overlook substantial opportunities to simultaneously improve public health. This approach highlights increased economic value for ecological restoration and supports more informed decision-making and investment in nature-based strategies that address climate change while delivering local benefits to ecosystems and communities.

## Graphical abstract

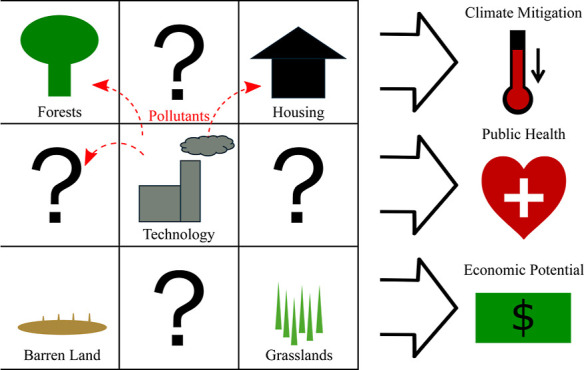

## 1 Introduction

Acknowledging the rapid increase of ongoing and predicted climate changes, carbon reduction strategies and targets are being set by entities across all scales. This includes businesses, governments, nongovernmental organizations, and even individuals. Of the actions being explored, nature-based solutions are increasing in popularity across climate discussions and sectors.^1^ As net-zero emissions and 100% renewable energy pledges are being made by almost all major corporations, like Apple, Coca Cola, Johnson + Johnson, and P& G,^2^ land-based responses are being identified as cost-effective and simple approaches for increasing carbon sequestration. These offer a negative emissions option for mitigating today’s carbon emissions and promoting future atmospheric stabilization.^3^ Based on a 2019 study, forests account for the sequestration of nearly 30% of global annual CO_2_ emissions.^4^ The potential for increasing this number varies as most studies focus across different scales, regions, or behavioral scenarios; however, it was estimated that the land use, land use change, and forest (LULUCF) sector could achieve as much as 20% of global mitigation targets, according to the Intended Nationally Determined Contributions submitted by parties of the United Nations Framework Convention on Climate Change (UNFCCC).^5^ Further, within the United States, carbon sequestration capacity has a reported potential to increase by 20% by planting trees on understocked forestland.^6^

Even though technological approaches can make significant reductions to carbon emissions inventories, there is currently no proven scalable carbon dioxide removal technology, which is assumed in all analyzed scenarios that meet the Paris Agreement goal of 1.5 °C warming relative to preindustrial levels.^7^ This technological limitation in the pursuit of “net-zero” highlights the significant role that nature-based solutions can play, offering negative emissions in varied scale based on current land cover use and availability for changes.^8^ Outside of global biogeographic regions, like comparing equatorial ecosystems and arctic tundra, carbon sequestration rates do not significantly vary based on location within most project boundaries, or even most country’s borders.^9^ Therefore, current locations of nature-based solutions are predominantly chosen based on land availability and social considerations.^8^ Land-related responses present a challenge of high transaction costs,^10,11^ however, this study suggests that cost-benefit analyses are inadequately accounting for the benefits of ecosystem restoration and land use changes when solely focusing on carbon uptake.

Utilizing ecosystems in the pursuit of carbon neutrality should consider ecological cobenefits, such as air quality regulation, to maximize the value of investments and land use. The hazards of exposure to criteria air pollutants like particulate matter, ozone, and sulfur dioxide include acute and chronic impacts on human health leading to lung, kidney, and cardiovascular diseases.^12,13^ Despite the success of many pollution control efforts, the World Health Organization (WHO) estimates that 4.2 million deaths every year are attributed to ambient air pollution.^14^ Nowak et al. (2014) conducted a nationwide analysis of air pollution (NO_2_, O_3_, PM_2.5_, and SO_2_) removal by trees in the U.S., estimating 17.4 million tonnes removed in 2010 with a corresponding economic value of 6.8 billion USD.^15^ This economic value is associated with the avoidance of over 850 incidences of human mortality and 670,000 incidences of acute respiratory symptoms due to reduced air pollution exposure, mostly in urban areas. Further, the impacts that pollution have on communities and individuals are not equally distributed and families with children, those of lower socio-economic status, and communities of color are more likely to have higher exposure.^16,17^ Similarly, the poor and vulnerable are predicted (with high confidence) to experience disproportionate climate change impacts that are associated with carbon and greenhouse gas emissions.^7^ Due to the inequitable cascading effects stemming from both criteria air pollutants and greenhouse gas emissions, sustainable solutions must include spatially explicit information that shows an understanding of the consequences and opportunities across the landscape and involved populations.^18–20^

Historically, engineering has evolved in a paradigm that ignores nature and implicitly assumes it to be a sink with infinite capacity. Research around techno-ecological systems and potential synergies challenges this paradigm and continues to evolve with conceptual frameworks and absolute metrics for sustainability,^21^ integration into life cycle assessment approaches,^22,23^ applications which define ecosystems as process and unit operations for air and water pollution control,^24,25^ and exploring the trade-offs across the food-energy-water (FEW) nexus.^26^ For sustainable industrial design and operation, both spatial and temporal dynamics have been explored. Recent spatially explicit analyses and design frameworks have been developed to explore the impacts of air pollution and land-based mitigation strategies, although health considerations have been excluded.^27,28^ Exploring dynamics, Shah and Bakshi developed a framework for design and operation of techno-ecological air pollution control systems in response to growth and seasonal intermittency of ecosystems, including social costs in their most recent work modeling health risks from air quality changes for a spatially homogeneous population.^29–31^

In alignment with the 2030 Agenda for Sustainable Development and United Nations Sustainable Development Goals (SDGs),^32^ sustainable engineering must expand to include methodologies and metrics that directly incorporate public health, disproportionate effects to society, and traditional economic metrics that dominate stakeholder interest. Projected climate impacts on harmful air pollutants were studied by Garcia-Menendez et al., concluding that climate change can significantly increase pollution and associated health effects and conversely, mitigating climate change can reduce these projected health impacts highlighting the intersection between climate and health risks.^33^ In response, we built upon our past optimization-based approaches for long-term climate action planning^8^ and spatially explicit industrial site design^28^ to develop a computational design approach that incorporates spatial health risk assessments and highlights the economic cobenefits of ecosystems in the pursuit of carbon neutrality and “net-positive” industries.^34^ Previous approaches inadequately address ecological cobenefits resulting in climate plans that lack spatial guidance for strategic nature-based solutions. Neglecting cobenefits also results in undervaluing the social and economic returns of ecosystem restoration and land-use changes.

This work provides a spatially explicit operationalization framework for including land-use changes (LUCs) into action-based climate plans that consider public health impacts and identifying both where and when LUCs should be implemented, leaving the “how” to local ecological experts and land management teams. A multiecosystem services approach promotes ecologically diverse landscapes with native species rather than monoculture plantations, which is why local knowledge is required for successful implementation. The “where” is based on reducing pollutant exposure and minimizing health-related incidences, like hospitalizations and mortality, while the “when” is dependent on budget constraints and ecological growth dynamics. A generalized design framework is described in the Methodology Section then a practical power generation case study is presented along with results that compare our previous spatially explicit approach with this cobenefit approach. The new insight from this approach results in higher economic valuation for nature-based solutions, promoting investments in land-use changes, and an increased focus on understanding the neighboring populations around industrial sites. Practically, it demonstrates the missed opportunities for more effective nature-based solutions when we only focus on one service, like carbon sequestration. This work does not address the challenges of measuring, monitoring, and reporting additional carbon sequestration, but it does acknowledge the challenge of high transaction costs by advocating for an increased return value against the investment.

## 2 Methodology

The TES Industrial Landscape Design Framework was presented in our previous work^28^ and Figure S3 gives an overview of the past work, while highlighting how this study extends the framework to include carbon sequestration and health risk assessments. Broadly, the framework combines models for both technological and ecological systems, describing module characteristics like cost, capacity, and pollution removal rates. Modules can be used to characterize traditional unit operations, like a selective catalytic reactor, or represent an ecological unit, like a hectare of forest. The modules are built through input information from either literature, like the EPA Air Pollution Control Cost Manual,^35^ or model simulations, such as dispersion modeling or species-specific ecological dynamics. Along with constraints specific to each module, the program must include case study-specific constraints that define the problem and limit the decision variables’ degrees of freedom. Example constraints include ensuring a minimum pollution uptake, operating within a given budget, or meeting a site’s production standard. The primary decision variables include the binary ecological decision variables (*E*_*n*,*i*_) representing whether and when afforestation occurs at each receptor location and the continuous variables (*S*_*n*,*j*_) representing the deployment levels of technological control options over time. Further, the objective function, or functions, of the optimization program must be defined such as minimizing private costs to industry, maximizing social benefits, or minimizing emissions rates. Additional details can be found in the Supporting Information.

Although this work provides a novel application of the TES Industrial Landscape Design Framework that focuses on the cobenefits of carbon management strategies, two main methodological contributions include incorporating health risk assessments and dynamic carbon sequestration models. By monetizing the social benefits of reducing public health risks and removing atmospheric carbon, these cobenefits were included within the economic objective function. Our initial development of the TES Industrial Landscape Design Framework focused on minimizing private costs of technological and ecological systems used in air pollution control techno-ecological systems. This approach solely accounted for the physical uptake performance [tonnes per $] of ecosystems versus technology without considering economic valuations of ecosystem services. The adjusted objective function and optimization formulation is provided in the Supporting Information along with other methodological details such as the pollution control technology models, ecological growth dynamics, and atmospheric dispersion and chemistry modeling parameters. The design framework relies on three main components: dispersion modeling (CALPUFF), health risk assessment (BENMAP-CE), and the optimization program.

### 2.1 Dispersion Modeling with CALPUFF

To develop spatially explicit data for the case study, we used the CALPUFF modeling system to represent regional surface characteristics, meteorology, and atmospheric chemistry, and to simulate land-use changes in order to quantify changes in pollutant concentrations and uptake. The geophysical model is built using land cover data from the United States Geological Survey (USGS) Land Use and Land Cover Composite Theme Grid (CTG) formatted data^36^ and elevation information from the USGS Digital Elevation Model.^37^ The modeling system was applied across a grid of receptors defining the spatial domain at which model outputs are evaluated. Due to limitations in the resolution of data available for conducting the health risk assessments, the spacing of the grid is 2.5 km between each node with 41 × 41 receptors (1681 total) centered around the pollutant point source. The total area of this grid spans 100 km by 100 km. Once the geophysical data, like elevation and land cover, is projected across the grid, meteorological data at both the surface and upper air sounding levels are processed to create a meteorological model. The upper air sounding data can be found from the National Oceanic and Atmospheric Administration Integrated Global Radiosonde Archive^38^ and the surface data can be found from the National Climatic Data Center Integrated Surface Hourly Data Archive.^39^

Atmospheric chemistry was represented using the MESOPUFF II chemical transformation module embedded within the CALPUFF modeling system, which simulates gas-phase reactions, secondary particulate formation, and dry deposition processes for sulfur, nitrogen, and particulate species. Background concentrations for ozone and other criteria pollutants were specified as boundary conditions to enable simulation of direct ozone deposition in the absence of a fully coupled photochemical grid model. The chemical background data and point source parameters of the study site are presented in the Supporting Information.

This case study focuses on afforestation efforts. Therefore, we simulated two scenarios of land use: one with the current land cover and another assuming that unconstrained areas (<50% developed land-use or water bodies) were converted to forest canopy. The two simulations define baseline and afforested scenarios, enabling ecosystem service benefits to be quantified as incremental changes in pollutant concentrations and dry deposition (pollution uptake) relative to current land cover. In this work, CALMETv6.5.0 and CALPUFF v7.2.1 were used to create rasterized values for pollutant concentration and dry deposition for both land cover states.

### 2.2 Estimating the Economic Value of Human Health Effects with BENMAP

Modeled concentration fields generated by the CALPUFF modeling system were used as inputs to the BENMAP-CE^40^ health impact assessment software to estimate spatially explicit public health outcomes associated with air quality changes. For both land-use scenarios, gridded concentrations of PM_2.5_ and ozone were extracted at receptor locations, converted to concentration-change fields, and mapped alongside BENMAP’s population and baseline incidence data sets. To align with epidemiological health functions, CALPUFF outputs were postprocessed to compute annual mean PM_2.5_ concentrations and annual averages of the daily maximum 8 h ozone concentrations at each receptor. Because the optimization framework operates at an annual time step and population and baseline incidence rates are treated as static, higher temporal resolution in the health assessment was not required for this study. Finally, because CALPUFF does not include a fully coupled photochemical model, estimated ozone-related health benefits reflect reduced concentrations associated with direct dry deposition, and may underestimate surface-level ozone impacts by neglecting ozone concentration responses to NO_*x*_ uptake.

Calculating the expected health effects of reducing air pollution requires determining which specific health incidences will be considered. Many epidemiology studies connect health incidences, like hospitalizations or mortality, with air pollution concentration levels. BENMAP includes a library of health impact functions and parameters based on recent literature. All the impacts included with the software are incidences associated with PM_2.5_ and ozone. Most epidemiology studies fit their findings to the following function

1 $$\Delta Y=Y_{0}\left(1-e^{-\beta \Delta C}\right)P$$

where Δ*Y* is the change in incidence, *Y*_0_ is the baseline incidence, β is the effect estimate from the epidemiology study, Δ*C* is the physical change in concentration, and *P* is the exposed population. Each health incidence is then assumed to have an economic value such as hospital admission costs or the value of a human life when considering mortality. The decrease in health incidences due to air quality improvements can then be valuated as social economic benefits through avoided health costs. The valuation of incidences is calculated using a linear function as follows

$${Ẑ}_{health}=\Delta Y\left({ẑ}_{incidence}\right)$$ 2

where ${Ẑ}_{health}$ is the total estimate economic value of avoided health impacts and ${ẑ}_{incidence}$ is a constant value dependent on a particular incidence.

The following case study will focus on “Mortality, All Causes” due to changes in both ozone and PM_2.5_ concentrations. The β values reported by Turner et al. are 0.001980263 (SD = 0.000500216) and 0.005826891 (SD = 0.000962763) for ozone and PM_2.5_, respectively, and is the only study in the BENMAP-CE suite that provides values for both PM_2.5_ and ozone in a study evaluating impacts across all ages.^41^ This study assumes an $8.7 million mean value for a human life, characterized by a Weibull distribution with parameters defined by the EPA Standard Valuation Functions for 2021 (available in the BENMAP-CE library with more details in the Supporting Information).^42^

This approach calculates economic impacts of health risks based on static states of land use. To represent the evolution of health benefits between the current land cover and an afforested land-use case, we modeled ecological growth dynamics using the U.S. Forest Service’s Forest Vegetation Simulator (FVS).^43^ Because dry deposition is the primary mechanism through which forest ecosystems reduce ambient PM_2.5_ and ozone concentrations in the modeling framework, changes in tree crown cover provide a physically meaningful link between static land-cover states and temporally evolving concentration reductions. Each simulation begins with barren land and simulates natural regeneration postplanting of sparse saplings. For dry deposition simulations, leaf area, or tree crown growth, is the significant input value of deposition calculations conducted by the CALPUFF modeling system. After simulating the adjusted tree cover over 80 years, we then normalize the rates by the maximum value and fit a polynomial regression function. This function ranging between 0 and 1 can then be used as a coefficient at different time steps to represent an adjusted social benefit valuation at each point based on growth dynamics. Figure S2 shows the dynamics of the species used in the case study.

### 2.3 Including the Social Benefit of Carbon Sequestration

With increasing attention to climate change, the value of carbon sequestration is increasing.^44^ Therefore, monetizing atmospheric carbon removal as a social benefit, or negative cost, is important for cost-benefit analyses of land-based carbon sequestration projects. Because most atmospheric dispersion models are used to trace criteria air pollutants, CALPUFF does not include carbon dioxide parameters in its source code. Therefore, we used the county-average carbon sequestration rates reported in iTree Canopy.^45^ However, like the deposition of ozone and PM_2.5_, the sequestration of carbon is not static and again the Forest Vegetation Simulator (FVS) is used. Instead of adjusted tree cover over time, we calculated the change of live carbon over time, both above and below ground. After normalizing the data, we defined the function using a fifth order polynomial regression. We used this specific regression for its fit, yielding an R squared value of 0.987. Again, Figure S2 has the tree growth simulation results. The data reveals two important observations: (1). Young forests emit, or respire, more carbon than they sequester and (2). The rate of carbon uptake increases as the forest matures until it reaches a peak. We expect polynomial regression analysis to fit other species growth scenarios simulated using FVS, however, simulations longer than 80 years may require different regressive approaches. In this study we assume the county average sequestration rate will be represented at peak growth which likely underestimates the additional carbon uptake of the land use changes.

Because each receptor represents a mixed land-cover area, we must ensure that the carbon sequestration is additional to existing forest sequestration. Under the LUC scenario, unconstrained receptors are assumed to become fully forested, requiring an explicit accounting of the current area of forest land cover across unconstrained receptors to calculate the resulting change of forest area. This value is then divided by the total area of forest land in the LUC scenario, yielding a fraction of forested LUC, *R*, ensuring that only additional sequestration is calculated.

Putting all these terms together, the carbon sequestration rate, χ with units [tonnes], is calculated as

$$\chi_{n}=U\left(V_{c}\right)R\overset{n}{\underset{h}{\sum }}\left(\overset{I}{\underset{i}{\sum }}E_{h,i}\right)\left(a{\left(n-h\right)}^{5}+b{\left(n-h\right)}^{4}+c{\left(n-h\right)}^{3}+d{\left(n-h\right)}^{2}+e\left(n-h\right)+f\right)$$ 3

where *n* is the time step, *i* is the receptor index, *U* represents all necessary unit conversions, *V*_c_ is the county average carbon sequestration rate, *R* is the fraction of forested LUC, *h* represents the age index, *E* is the ecological binary decision variable, and *a* through *f* are regression coefficients. It is important to note that *E* is a matrix of binaries where a column for receptor *i* only equals 1 if a particular location is chosen for restoration in the optimal solution. If restored, it only equals 1 at the year of initial planting. To calculate the total sequestration at a particular time step (*n*), eq 3 checks each preceding year (*h*) and sums up the number of receptors that were planted at that time step. The age of restoration is calculated by (*n* – *h*), which is used in the growth dynamics equation to yield a value between 0 and 1. The sum of receptors and the growth ratio are multiplied by each other like a weighted average because the sequestration rate is dependent on the maturity of the restoration. The age index, *h*, is then iterated until it reaches the time step of calculation (*n*), summing up the sequestration of restoration locations with various ages. Then the county average carbon sequestration rate, fraction of forested land changes, and unit conversions are multiplied to achieve the appropriate units for χ_*n*_.

Because the social economic value for carbon sequestration is not dependent on population exposure, the conversion between physical units and economic valuation is a linear function, as follows

4 $${Ẑ}_{carbon}={ẑ}_{carbon}\sum_{n}^{N}{\mathrm{\chi ̃}}_{n}$$

where ${\mathrm{\chi ̃}}_{n}$ represents net carbon removal and ${ẑ}_{carbon}$ is the assumed value of carbon removal per unit of mass. Because the case study presented in Section 2.4 imposes carbon neutrality as a system-level constraint, eq 4 captures only the additional societal benefits associated with this net removal. The cost of carbon, or social benefit when considering sequestration, was reported by the Biden Administration at $56.2 per tonne ($51/ton) in 2021.^46^

### 2.4 Case Study: Hamilton County, Ohio

The case study presented in this work focuses on a coal-fired electric power station in Hamilton County, Ohio (outside Cincinnati). The study is designed to achieve carbon neutrality while exploring how the inclusion of health risk assessments impact where nature-based solutions are optimal and the overall valuation of ecosystems and their services. The core purpose of this application is to highlight our ecological cobenefit framework which maintains carbon neutrality and reduces health risks within a spatially explicit TES industrial site design. To do this, we create a techno-ecological system boundary that includes air pollution control technology and land-use changes as an ecological solution, as outlined in Figure 1. The scope of this study is limited to the downstream air pollution removal systems, meaning that the design and economics associated with the coal-combustion and electricity production will be outside of our focus. We will consider the following site emissions: NO_*x*_, primary PM_2.5_, and CO_2_, and three associated technologies, a selective catalytic reduction reactor (SCR), a baghouse filter (BHF), and a carbon capture and storage unit (CCS). Like CO_2_’s neutrality target, PM_2.5_ and NO_*x*_ are also constrained to meet a minimum total pollutant uptake rate across the techno-ecological system. Notably, SO_2_ emissions are modeled as precursors to secondary particulate formation, and primary PM_10_ emissions are included as directly emitted particulate matter. The energy consumption and associated emissions of the CCS system are not explicitly modeled and the carbon uptake is assumed to be net CO_2_ benefits. Details on the facility, point source emissions parameters, mitigation targets, and the air pollution control models are provided in the Supporting Information.

**Figure 1:**
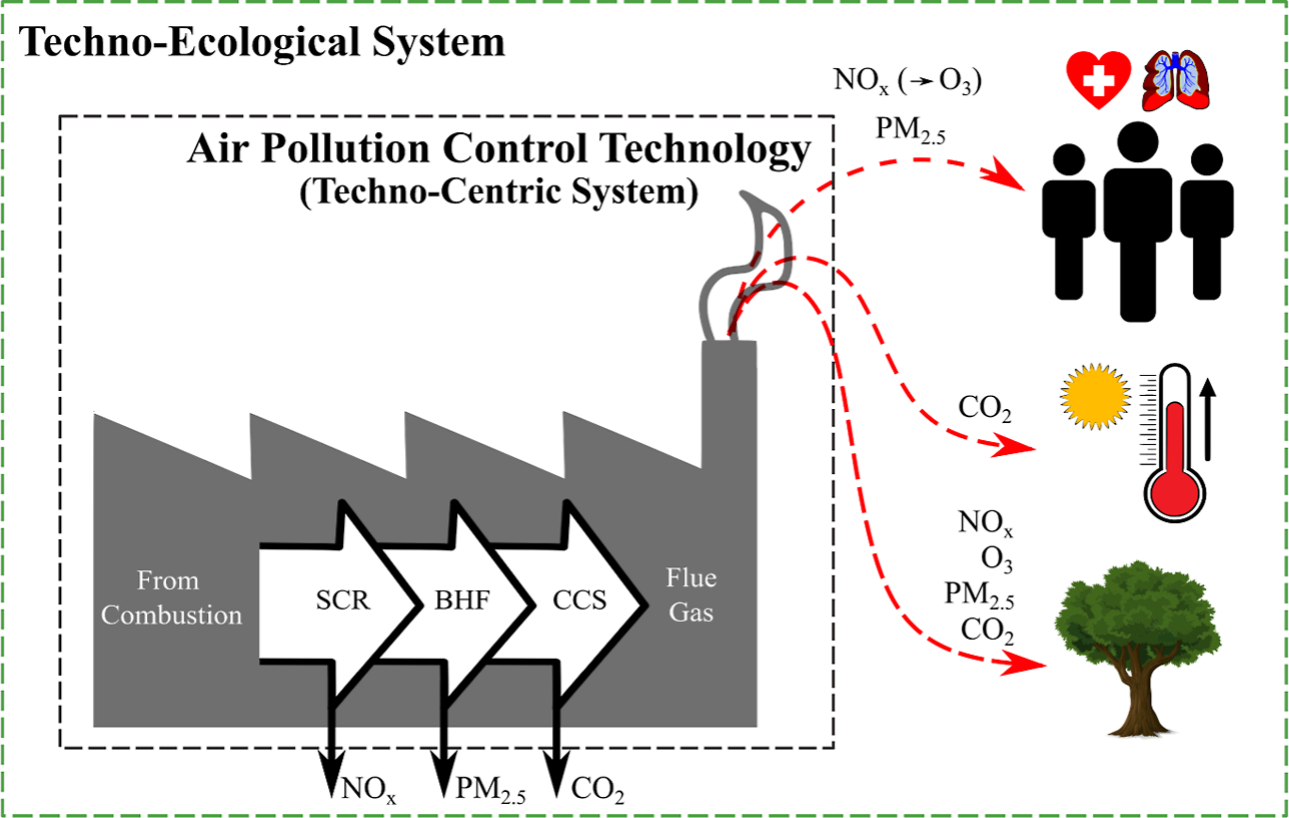
Techno-ecological system boundary for the case study. Land-use changes provide ecosystem services that mitigate health and climate impacts through the physical uptake of NO_*x*_, PM_2.5_, and CO_2_ via dry deposition and carbon sequestration. SCR: selective catalytic reduction reactor; BHF: baghouse filter; CCS: carbon capture and storage unit.

Despite the physical uptake constraints of PM_2.5_ and NO_*x*_, health impacts are only evaluated for pollutants with established epidemiological concentration–response relationships and population exposure metrics, namely PM_2.5_ and ozone. Although ozone is not directly emitted by the facility, background ozone concentrations are included to capture health benefits associated with direct dry deposition to forest canopies. Carbon mitigation, the other ecosystem service considered in this study, is economically valued as a social benefit regardless of whether sequestration occurs through CCS or land-use change, reflecting the absence of enforceable carbon neutrality policies.

The region near Hamilton County, Ohio contains the Ohio River and a mixture, of developed, forest, and agricultural land cover. To promote native and ecologically diverse tree species in afforestation and ecological efforts, we modeled forest stands with an even mix of three of the most popular species currently living across Ohio (yellow-poplar, sugar maple, and white ash).^47^ As shown in Figure S2, these metrics are used to represent both the change of carbon sequestration and dry deposition rates of NO_*x*_, ozone, and PM_2.5_ over time. The county average carbon sequestration value reported by the iTree Canopy tool is 11.2 tonnes CO_2_ per hectare per year, which was assumed as the maximum value for all restored forest area.^45^ The cost for afforestation was set to $376 per acre, representing a planting-only cost derived from Kroeger et al.^48^ under a seedling-based afforestation scenario. This value excludes land acquisition costs and is based on the high-cost planting assumptions in that study, including $0.60 per hardwood seedling, $85 per acre for machine planting, and $115 per acre for site preparation (double disking), applied at a base planting density of 730 seedlings per ha.

This study evaluates five design cases over a 60 year planning horizon (case labels in italics), each designed to isolate different aspects of techno-ecological system performance. The Tech Only case represents business-as-usual operation using technological controls alone, with no land-use change (LUC). The TES case applies the TES Industrial Landscape Design Framework,^28^ optimizing the system based on the physical pollutant uptake performance of ecological and technological controls without monetizing social benefits. The TES+ case extends this framework by incorporating the economic value of avoided health and climate impacts into the objective function, allowing both the scale and spatial distribution of afforestation to respond to social valuation.

To isolate the effect of spatial allocation independent of total afforested area, the TES + $ case applies the same objective function as TES+ but constrains the total afforested area (i.e., total LUC extent) to match that of the TES case. This allows for direct comparison of spatial prioritization under identical levels of ecological investment. Finally, the CO_2_-$ case represents a counterfactual scenario that prioritizes carbon neutrality without incorporating health-based valuation, constraining total afforested area to match TES + $ while minimizing health cobenefits. Together, TES + $ and CO_2_-$ define upper and lower bounds on cobenefit performance under carbon-neutral land-use strategies with and without spatial health prioritization. All cases that include ecological interventions impose an annual budget of $10.4 million USD (5% of business-as-usual operating costs).

The optimization framework was implemented as a mixed integer linear program, solved using the Gurobi Optimizer version 13.0.0 with the JuMP package in the Julia language. The program includes 720 continuous variables and 91,440 integer variables and solves between 2 and 3 min per run when using Windows 11 operating system on a 11th Gen Intel­(R) Core­(TM) i7 processor at 3.00 GHz with 16.0 GB RAM.

## 3 Results and Discussion

The biggest value of this work is the production of maps that provide a strategy guiding where to plant trees. Figure 2 shows three maps of input and intermediate data: (a) population variation within the model’s spatial boundaries, (b) the potential change of ozone related to land use changes, and (c) the potential change of particulate matter related to land use changes. Because the area is constant between each receptor, or pixel, the population and population density values only differ by the conversion of units (number versus number per area). The change of concentration shown in Figure 2b,c are direct outputs of CALPUFF and the range represents concentration reductions, or the absolute value of Δ*C*, so that darker red shows the higher change in concentration associated with the simulated landscape changes. These maps are derived solely from the dispersion modeling and represent the underlying spatial response of pollutant concentrations to land-use change, independent of the optimization scenarios, which determine the final selection of afforestation locations. The city of Cincinnati lies east of the manufacturing site, shown as the areas of higher population and lower potential changes in ozone and particulate matter concentrations in Figure 2. Because ozone is not directly emitted from the point source, modeled ozone concentration changes are most strongly related to the availability of land cover for afforestation, favoring areas with substantial grass, agricultural, and barren land cover and disfavoring highly developed or already forested areas. In contrast, PM_2.5_ concentration changes exhibit a stronger dependence on distance from the point source, producing a near-radial pattern with the largest reductions occurring north of the facility along the dominant wind direction and diminished impacts within urban areas constrained from land-use change. This case study assumes agricultural land is available for LUC as a best-case scenario for carbon removal, but disregards the competition for land use between food production and forestry-focused carbon sequestration. This assumption represents an upper-bound scenario for ecological deployment and does not imply that all agricultural land is economically or practically available for conversion.

**Figure 2:**
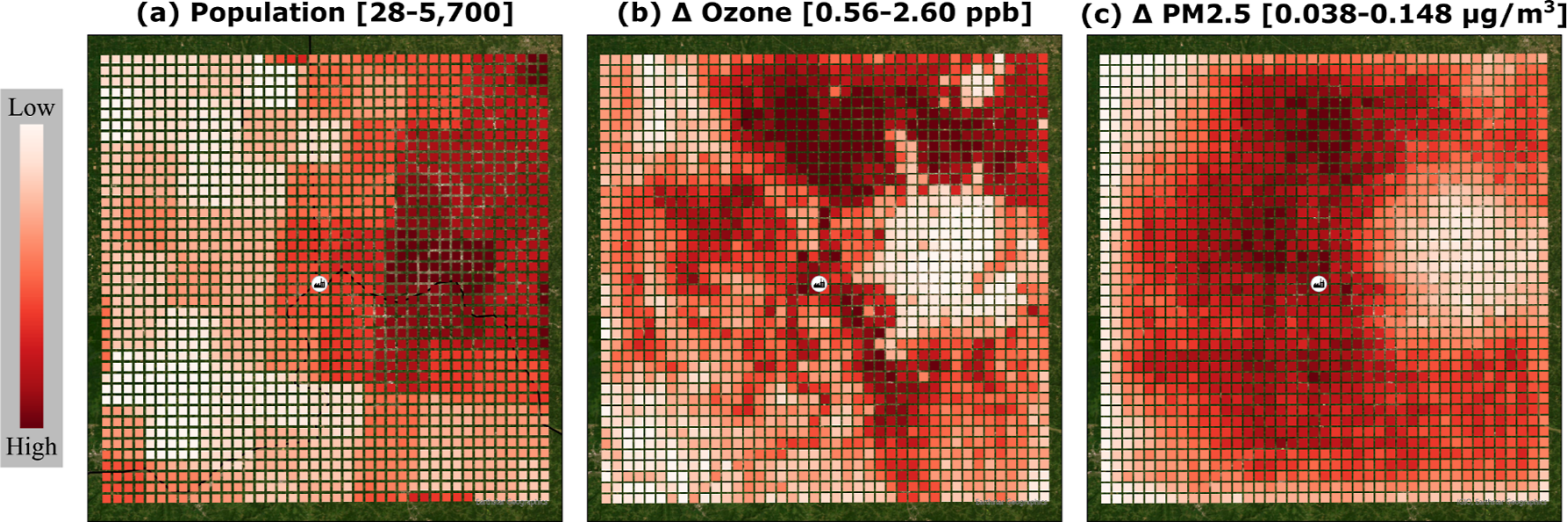
Intermediate results from the dispersion modeling: (a) population distribution around the facility, (b) potential change of ozone concentration, and (c) potential change of particulate matter (PM_2.5_) concentration under full afforestation of unconstrained areas. The grid spans a 100 km × 100 km domain with the power plant centered. Values in (b,c) represent the magnitude of concentration reductions (absolute values of Δ*C*), such that darker red indicates greater potential improvement in air quality.

Comparing the carbon neutrality design scenarios defined in Case Study: Hamilton County, Ohio, Table 1 highlights the optimization results through annualized values of the economic and physical benefits associated with each scenario. The reported uncertainty reflects the propagation of uncertainty from the epidemiological effect estimates, utilizing the distributions associated with the β value in eq 1 and the Weibull distribution associated with the value of a human life. This uncertainty influences the estimated magnitude of averted mortalities and associated social benefits but does not significantly alter the qualitative comparison between design scenarios or the dominant spatial patterns of land-use change shown in Figure 3, with only minor variations observed under sensitivity analyses. Additional sources of uncertainty, including dispersion modeling, ecological growth dynamics, and pollutant uptake rates, are not explicitly quantified in this analysis due to data and modeling limitations.

**Table 1:** Comparison of Optimization Results Across Five Design Scenarios Described in Section 2.4 ^a^

	tech only	TES	TES+	TES + $	CO2-$
private costs [USD]	225 million	160 million	162 million	160 million	160 million
additional averted	0	8.3	29.0	27.8	0.73
mortalities		(0.7, 23.2)	(2.5, 81.0)	(2.4, 77.6)	(0.07, 2.04)
carbon uptake [Mt]	6.06	6.81	7.03	6.81	6.81
biogenic sequestration	0	45.8	47.6	45.8	45.8
[% of total CO_2_ uptake]					
total social benefits	341	455	647	624	389
(million USD)		(389, 585)	(416, 1100)	(403, 1058)	(383, 400)

a Tech Only represents business-as-usual without land-use change (LUC). TES includes LUC and optimizes for the lowest cost techno-ecological system to meet minimum pollutant uptake constraints, with social benefits evaluated post-optimization. TES+ incorporates the monetized social benefits of climate and health risk mitigation directly into the optimization objective. TES + $ applies the TES+ framework under a LUC budget constraint matching the total restored area in TES. CO_2_-$ represents a carbon-focused strategy with the same LUC budget but minimal health risk reduction. All values are annualized averages over a 60-year simulation, with 95% confidence intervals (from health risk assessment) shown in parentheses.

**Figure 3:**
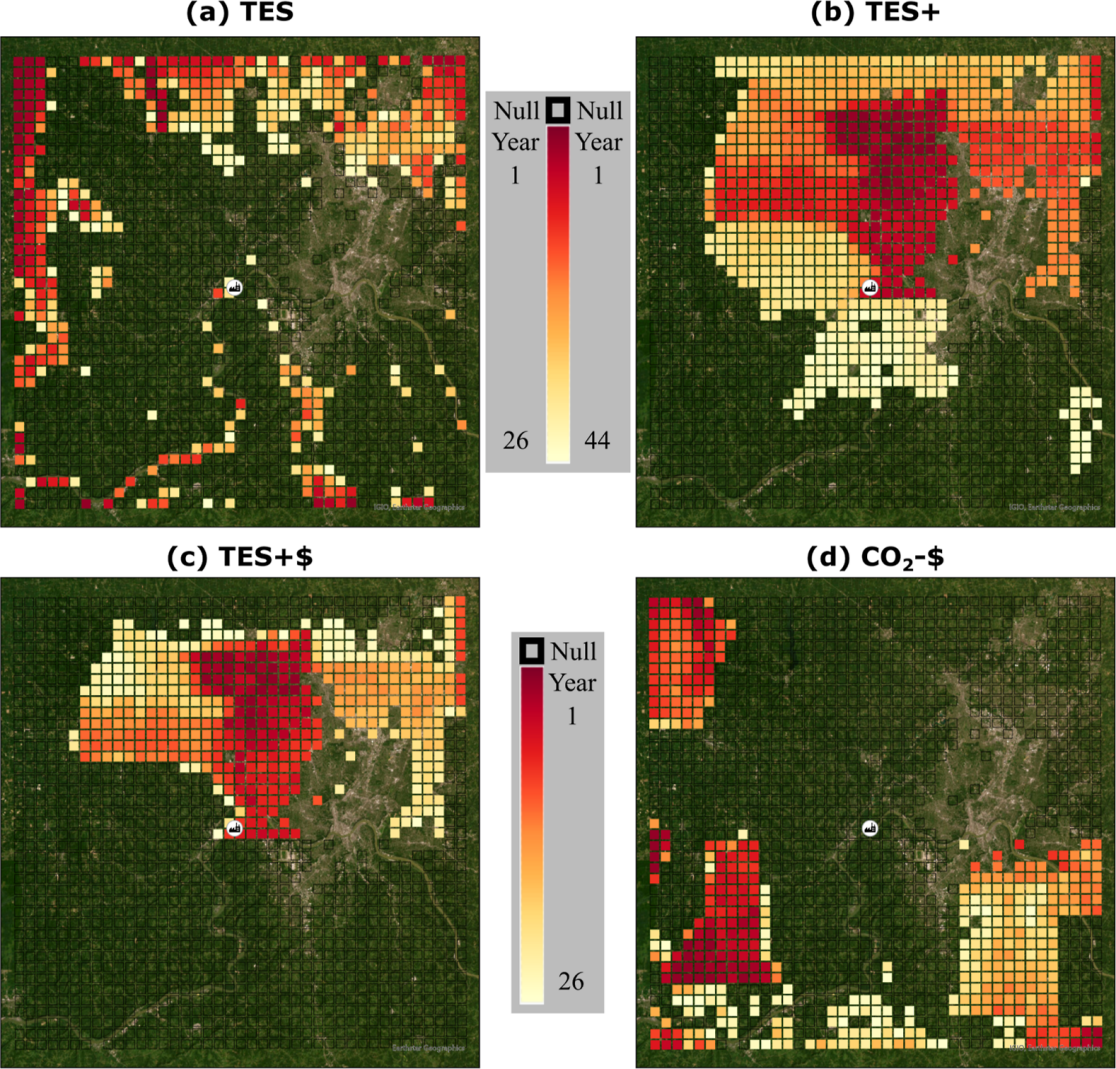
Spatiotemporal maps of land-use change (LUC) solutions for Hamilton County, OH, comparing four design scenarios described in Section 2.4: (a) TES, which optimizes solely on physical pollutant uptake without social benefit valuation. (b) TES+, which incorporates spatially explicit health risk assessment and social costs of carbon into the optimization. (c) TES + $ and (d) CO_2_-$, which match the total LUC area of (a) and represent the best- and worst-performing spatial distributions for social benefits, respectively. Colors represent the year in which afforestation is selected at each location by the optimization model over the 60 year planning horizon, with earlier years (red) indicating higher-priority locations and later years (yellow) indicating lower-priority locations. Receptors shown as empty black squares represent available land that is not selected in the optimized solution. The larger spatial extent of LUC in (b) relative to (a) reflects the inclusion of health and climate benefits in the optimization.

Notably, every scenario that includes LUCs shows improvement from Tech Only, or the business as usual case, in both private cost and social benefit objectives. This highlights potential “win–win” solutions for techno-ecological systems versus solely technological approaches. Including 60 years of ecosystem growth, the investments in LUCs and restoration cut the costs for air pollution control by 28–29%. This is largely because planting trees is cheaper than current technological carbon capture and storage systems, assuming land availability. For the Tech Only case, carbon neutrality is achieved at an uptake rate of 6.06 Mt CO_2_/yr, resulting in a social benefit valuation of $341 million annually. This defines the minimum required CO_2_ removal across all scenarios. Cases that include land-use change can exceed this level, and in Table 1, biogenic sequestration (%) indicates the fraction of total CO_2_ uptake attributable to land-use change rather than technological capture.

In the TES scenario, where health impacts are not included in the objective function, the overall scale of land-use change is driven primarily by carbon sequestration benefits, as the physical uptake of NO_2_ and PM_2.5_ provides comparatively limited reductions in the operation of the SCR and BHF technologies (see Figure 4c). This reflects the underlying ecological processes, where CO_2_ is sequestered and stored throughout forest biomass above and below ground, while PM_2.5_ and NO_*x*_ are removed only through surface-level dry deposition to leaf area. Because each case meets the annual removal rate for each pollutant, as explained in Section 2.4, the Tech Only case is considered the baseline for averted mortalities and the TES approaches offer additional averted mortalities through LUCs.

**Figure 4:**
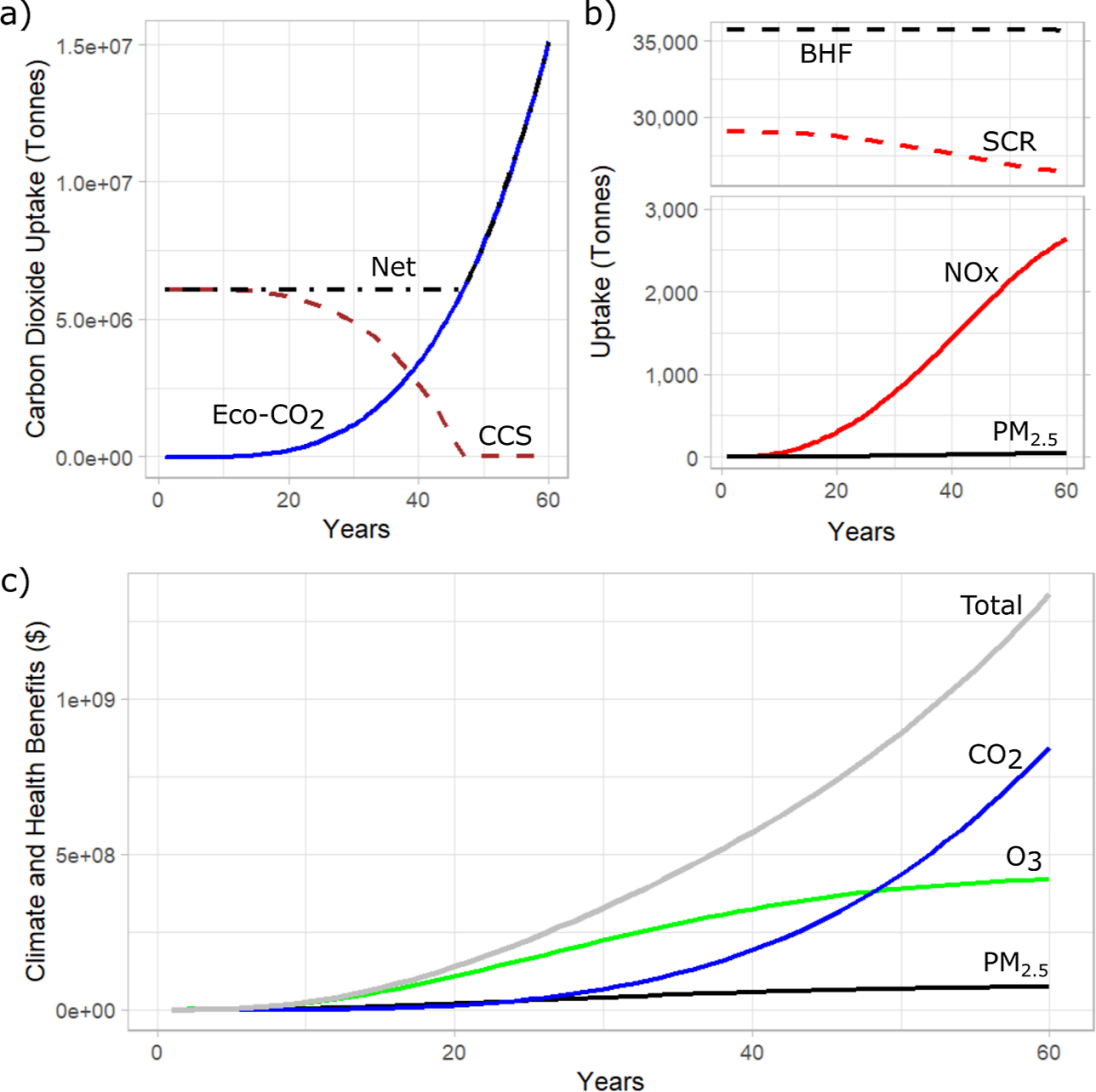
Temporal dynamics of techno-ecological system performance and monetized social benefits for the TES+ design scenario. (a) Trade-off between technological carbon capture and storage (CCS) and biogenic carbon sequestration (Eco-CO_2_) in meeting the carbon neutrality constraint. (b) Ecological deposition of NO_*x*_ and PM_2.5_ and the corresponding operation of pollution control technologies, the selective catalytic reactor (SCR) and baghouse filter (BHF). (c) Monetized climate and health benefits associated with ecological uptake of CO_2_, O_3_, and PM_2.5_, and their contribution to total social benefits.

Comparing the TES+ scenario with TES, the valuation of social benefits in the objective function led to an increased scale of LUC and a 247–257% increase in averted mortalities for only a 1.3% increase in private costs. Including the monetized valuation of health and climate benefits in the objective function, the overall increase of social benefits ranges from 7 to 88%, with a mean of 42%. The range of percent increase values are calculated at the 2.5 percentile, mean, and 97.5 percentile values shown in the table.

Because the carbon sequestration benefits are not spatially dependent, they are related to the overall land area that is afforested and restored. This is seen as the cases TES and TES + $ are compared. The two cases have equal carbon sequestration rates because they have the same total area of LUCs. The TES + $ scenario is included to look at our methodological advantages of including health risk assessments given the same LUC budget allowance. For nearly the same private costs, averted mortalities increase by 234–243% and an overall social benefit improvement of 4–81%, with a mean of 37%. The slightly lower private cost in TES relative to TES + $ reflects small differences in how the minimum uptake constraints are met, allowing marginal reductions in required technology operation (and associated operating costs), particularly for the SCR.

Finally, we included the scenario CO_2_-$ to look at the range of public health benefits, or averted mortalities, that can occur if we implement spatially arbitrary LUCs while maintaining the carbon mitigation target. This “worst case” scenario sequesters as much carbon as the TES and TES + $ cases, but only provides 2.6–2.9% of the averted mortalities as the “best case” scenario with the same budget, TES + $. By excluding health risk assessments in the implementation of nature-based solutions for climate mitigation, one risks forfeiting up to 97% of the potential public health benefits (averted mortalities) achievable with the same investment. It should be noted that all TES-based scenarios (including LUC) overachieve carbon neutrality operations by sequestering more than is emitted, as each value is greater than the 6.06 megatonnes of CO_2_ emitted by the facility.

Improvements between design scenarios are rooted in the inclusion of spatially explicit health risk assessments. With the simulation of growth dynamics, the LUCs can be shown in spatiotemporal maps. These maps are shown for the cases that include LUCs in Figure 3. The larger extent of afforestation (LUC) in the TES+ case (Figure 3b) compared to TES (Figure 3a) results from the modified objective function in TES+ (eq 2 in the Supporting Information), which increases the economic return on land-use change investments and favors larger restoration scales. Ecosystem service benefits accumulate year after year without further investment, but require time to realize these returns on investment. By only considering physical uptake rates, the TES scenario (Figure 3a) requires approximately 34 years of ecological growth before land-use change is selected by the optimization as the lower-cost alternative relative to technological options, limiting implementation to the first 26 years of the model time span. On the contrary, TES+ requires only 16 years of ecological growth before land-use change is selected as the lower-cost alternative within the optimization. The timing in Figure 3 reflects the year in which afforestation is selected at each location by the optimization model and emerges from the interaction of ecological growth dynamics, costs, scenario-specific objective functions, and budget constraints on land use change that require prioritization over time.

Spatially, the pollutant uptake values of NO_*x*_ and PM_2.5_ that drive the distribution of the TES solution are primarily governed by existing land cover and, to a lesser extent, pollutant dispersion from the point source. Land cover is a dominant factor because each receptor represents a 2.5 km × 2.5 km area, while the Land Use and Land Cover Composite Theme Grid (CTG) data^36^ are resolved at 200 m × 200 m, resulting in 156 land-cover cells aggregated within each receptor. The fraction of existing forest and vegetation within a receptor therefore strongly constrains the capacity for additional ecological growth and dry deposition. Pollutant dispersion contributes secondarily, as dry deposition of PM_2.5_ and NO_*x*_ depends on surface-level concentrations and meteorological conditions.^49^ In this region, the dominant wind direction is between north and northeast, leading to moderately higher deposition potential downwind of the facility. Consequently, the TES solution exhibits a dispersed pattern, favoring rural areas with agricultural and grassland cover available for afforestation, particularly along the northern and western boundaries of the modeling domain. A visible artifact of the receptor resolution appears along the Ohio River, where mixed land-cover classification allows receptors with less than 50% water cover to be treated as unconstrained, resulting in afforestation of areas that partially represent water surfaces. Across Figure 3, constrained or unavailable areas are indicated by the absence of black squares.

When social benefit values are introduced, population exposure becomes the dominant spatial driver. Because avoided health impacts scale directly with exposure (eq 1), incorporating health risk assessments shifts land-use changes toward areas with higher population density. Comparing TES and TES + $ in Figure 3a,c, the most notable difference is the emergence of more clustered solutions in TES + $. These clusters occur around the urban hub of Cincinnati, where population density is highest and land remains unconstrained (<50% developed). By contrast, the CO_2_-$ scenario (Figure 3d) prioritizes locations that are spatially ineffective for reducing pollution exposure, with land-use changes concentrated in areas of low population (see Figure 2a).

Although carbon uptake and climate benefits do not contribute to the spatial outcomes of the design framework, they do impact the scale of LUC as they compete economically with early stage carbon capture technologies to meet neutrality requirements and provide increasing social benefits over time. Without budget constraints, all feasible land-based projects would be implemented in the first year to maximize return on investment. However, budget constraints and ecological growth dynamics impact how the techno-ecological system must dynamically respond by adjusting the operation of the air pollution control technologies to meet pollutant uptake constraints. Figure 4 illustrates the temporal dynamics of technological and ecological units and the corresponding climate and health benefits featured in the TES+ design scenario (mean). Figure 4a compares biogenic carbon sequestration (Eco-CO_2_) with technological carbon capture and storage (CCS) in meeting the carbon neutrality constraint. Through Year 13, neutrality is achieved entirely through CCS as the forest stands mature and switch from net emission of CO_2_ to sequestration. At this point, biogenic sequestration grows exponentially through the remaining duration of the model surpassing the contributions of CCS at Year 39 and achieving neutrality by Year 44, eliminating the need for CCS. Past Year 44, the system achieves “net-positive” impacts on climate, or negative carbon emissions, for the remaining years of the simulation. Continued planting through Year 44 sustains the exponential increase in biogenic carbon uptake, even though individual forest stands are expected to approach peak sequestration rates at approximately 60 years of age (Figure S2). This highlights how staggered implementation of land-use changes can sustain ecosystem services across time and is a foundational practice of sustainable forestry.

Unlike carbon capture, ecological uptake of criteria pollutants does not substantially displace technological controls, even at these large spatial scales. Figure 4b highlights the techno-ecological operation for maintaining NO_*x*_ and PM_2.5_ pollution uptake rates. After 60 years, the SCR continues to operate at 90.9% of its initial capacity and the BHF at 99.9%, showing that private cost reductions for the facility’s mitigation of NO_*x*_ and PM_2.5_ are not significant.

Figure 4c shows the dynamics of the valuated climate and health benefits over the 60 year simulation. In the early and midterm years, ozone-related health benefits dominate the total social benefits as tree crown cover increases faster than embodied carbon (Figure S2) before stabilizing, as observed toward the end of the simulation. At Year 49, the exponential growth of the climate benefits surpasses the flattening ozone health benefits. At their peak, PM_2.5_-related health benefits still contribute approximately $72 million USD per year, despite comparatively low physical uptake rates. This underscores the disproportionately high economic value of PM_2.5_ removal due to its severe health impacts. By explicitly incorporating health impacts into the valuation of ecosystem services, nature-based solutions generate meaningful social benefit returns much earlier than climate benefits alone, effectively accelerating the growth of total benefits over time.

Given the role of carbon valuation in the objective function, we evaluated the sensitivity of these results to the assumed social cost of carbon (SCC). In this study, a constant SCC is used to isolate the effects of spatially explicit health cobenefits and ecological dynamics, while avoiding the introduction of additional scenario-dependent assumptions. To assess the robustness of this choice, a low-value SCC case of $11/tCO_2_ was evaluated, corresponding to the lower bound of EPA estimates.^50^ While the total extent of afforestation decreased by approximately 40%, the spatial prioritization remained unchanged, with all selected locations forming a subset of those identified under the baseline case. This indicates that SCC primarily influences the scale of land-use change, whereas spatial allocation is driven by health cobenefits and pollutant exposure patterns. Notably, SCC trajectories reported in policy analyses typically increase over time due to rising marginal damages, suggesting that the use of a constant SCC may underestimate the long-term value of carbon sequestration. Further work may incorporate time-varying SCC trajectories; however, the results of our study indicate that such refinements are unlikely to alter the spatial prioritization of land-use decisions.

Although constraints have been added to make the design scenarios more practical, several assumptions underlie the results presented in this work. Most notably, all land within the region with less than 50% developed area is assumed to be available for land-use change, neglecting land-use competition between agriculture for food and forests for air quality regulation. Further, questions of land ownership and allocation of credit for ecosystems services and social benefits are not addressed. Who pays the bill and who gets credit? Proper allocation and proven additionality of benefits remain significant barriers to implementation under existing markets and policy frameworks, particularly for pollutants external to emerging carbon markets. From a regulatory perspective, this work assumes that ecosystem-based pollutant uptake can substitute for technological pollution controls. However, such equivalency is not defined within current regulatory frameworks, highlighting a key barrier to industrial investment in land-use change while potentially shifting incentives toward other stakeholders, such as insurance companies or local governments.

Several modeling limitations also motivate clear directions for future work. Ozone dynamics are simplified relative to the full complexity of nonlinear interactions among NO_*x*_, VOCs, and photochemical processes, and improving the accuracy of ozone simulation will require coupling with complete photochemical models, like CMAQ or CAMx. In addition, this study assumes that concentration changes at each receptor are driven by localized changes in deposition flux and does not resimulate atmospheric dispersion for every land-use change configuration evaluated during optimization to capture feedbacks from neighboring land-use changes. This approximation reflects practical limitations in coupling CALPUFF with large-scale optimization frameworks and could be addressed in future work through reduced receptor or temporal dimensionality, or by introducing parametrized land-use decision variables, each of which introduces its own trade-offs.

To validate these models and design frameworks, industrial partnerships will be crucial in the realization of pilot-scale applications, building on precedents such as Dow Chemical’s Seadrift Constructed Wetlands.^51^ Future work should also consider ecosystem disservices such as allergen exposure, VOC emissions from afforestation, land and water use competition, aesthetic impacts, and evapotranspiration effects.

Beyond the case-specific results, this study highlights several broader insights for the design of sustainable industrial systems and nature-based climate strategies. First, the results demonstrate that spatial heterogeneity in population exposure and pollutant dispersion can strongly influence the distribution of health-related ecosystem service benefits, leading to distinct spatial patterns of optimal land-use change. Approaches that evaluate afforestation or restoration solely on carbon sequestration potential may therefore overlook significant opportunities to improve public health and increase overall societal value. Second, the framework shows that carbon and health cobenefits play distinct roles in decision-making, with carbon valuation influencing the overall scale of land-use change and health impacts driving spatial prioritization. This separation indicates that incorporating cobenefits does not simply increase total value, but fundamentally alters where interventions are most effective.

More broadly, the techno-ecological systems framework presented here provides a generalizable decision-support tool for integrating ecological processes into industrial design and climate planning. By combining process-based dispersion modeling, health risk assessment, and optimization, the approach can be applied across a range of sectors and regions to identify locally optimal strategies for pollution mitigation and carbon management. While this study focuses on a single power generation facility, the underlying methodology is transferable to other industrial systems, land-use contexts, and ecosystem services, supporting more informed, spatially explicit investment in nature-based solutions. Integrating spatially explicit health valuation into techno-ecological system design reframes nature-based solutions from passive carbon sinks into operational processes that complement engineered technologies, improve public health, and deliver substantial societal benefits with minimal cost trade-offs. This work highlights a pathway toward more effective and equitable sustainability strategies by coupling engineered systems with ecological processes to achieve climate and public health goals simultaneously.

## Supplementary Material



## Data Availability

The optimization model code and all associated input data files used in this study are available on Zenodo at the following link: 10.5281/zenodo.18262658. In addition, the repository includes preprocessing codes for the CALMET modeling system, including scripts for generating SURF.DAT and UP.DAT files from publicly available surface and upper-air meteorological data. These preprocessing tools are compatible with current data formats provided by the National Oceanic and Atmospheric Administration (NOAA) archives.

## Notes

Details of the optimization framework, additional model formulation, input data sets, and supplementary figures and tables (PDF)
