# Supplementary material for: Where to Plant Trees? Designing Net-Zero Industrial Landscapes that Promote Public Health
Source: Environ Sci Technol. 2026 Apr 23;60(17):12906–17. doi: 10.1021/acs.est.6c00851 (PMC13151038; doi:10.1021/acs.est.6c00851)
Supplement: Supplementary file 1 [file es6c00851_si_001.pdf]

Supporting Information

Where to Plant Trees? Designing Net-Zero Industrial Landscapes that Promote Public Health

Michael Charles*[a] and Bhavik R. Bakshi[b,c]

[a] Department of Biological and Environmental Engineering, Cornell University, Ithaca, NY 14853, USA;
[b] William G. Lowrie Department of Chemical and Biomolecular Engineering, The Ohio State University, Columbus, OH 43210, USA;
[c] School for Engineering of Matter, Transport and Energy, School of Sustainability, School of Complex Adaptive Systems, Arizona State University, Tempe, AZ 85281, USA
*Corresponding Author and Lead Contact; Email: mtc58@cornell.edu, Tel: +1-607-254-3232, Address: 111 Wing Dr., Ithaca, NY 14853

Contents

Pages: S1 - S8
Figures: S1 - S3

S1 Case Study Parameters: Hamilton County, Ohio

The electric power plant has a capacity of 1,444 MW and produced 5.4 million MWh in 2018.[1] Emissions and data comes from the EPA's National Emissions Inventory dataset[2] and the total annual emissions yielded 15,174 tonnes of SO_2, 8,721 tonnes of NO_x, 861 tonnes of PM_{10}, 707 tonnes of $PM_{2.5}$, and 6,060,000 tonnes of CO_2. These values were assumed to be emitted at a constant rate over a year. In 2018, the power station produced 5,409,444 MW-hr of electricity.[1] We modeled the power station as a single point source with a stack height of 243.8 m, a diameter of 7.163 m, at a temperature of 328 K, with an exit velocity of 20.78 m/s.[2] The final data needed for dispersion simulation is the background concentration data of the region for the modeled pollutants.

Now because the reported emissions of the plant already account for the existing pollution removal technology, an important assumption must be made: the overall efficiency of technological equipment. We assumed a removal efficiency of 80% of NO_x for the SCR and a 95% efficiency of $PM_{2.5}$ for the BHF. These efficiencies are conservative estimates based on ranges reported in literature.[3,4,5] For CO_2, no existing CCS unit was reported. These removal rates were used to back-calculate the annual minimum removal rate of each pollutant, Q^*. This minimum removal rate is assumed to replicate the current state of pollution control technology, which equals the reported emissions rate multiplied by $\frac{e_n}{1-e_n}$ where e is the removal efficiency of pollutant n. Details of all Air Pollution Control Units are found in Section S6.

Meteorological inputs were developed using surface and upper-air observations spanning January 1–December 31, 2024. Hourly surface meteorological data were obtained for station 93814 at Cincinnati/Northern Kentucky International Airport (CVG), reported in Eastern Standard Time (UTC–5).[6] Upper-air soundings were obtained at 12-hour intervals (00Z and 12Z) from station USM00072426 (Wilmington, OH) and processed in UTC, consistent with CALMET requirements.[7] Missing upper-air sounding data were patched using the last recorded value.

Background concentrations for SO_2, NO_x, $PM_{2.5}$, PM_{10}, and ozone were derived from U.S. EPA Air Quality System (AQS) monitoring data using daily datasets downloaded from the EPA outdoor air quality data portal.[8] Annual mean concentrations were calculated and used as base background concentrations for the MESOPUFF II chemical transformation module within CALPUFF.[9] Using annual arithmetic mean concentrations derived from EPA AQS data for calendar year 2024, the background concentrations simulated were 13.82 µg SO_2/m^3, 35.72 µg NO_x/m^3, 16.10 µg PM_{10}/m^3, 7.40 µg $PM_{2.5}/m^3$, and 90.64 µg O_3/m^3 (46.3 ppb, assuming 25C, 1 atm), with O_3, $PM_{2.5}$, and SO_2 obtained from the Colerain AQS site (39-061-0010) and NO_x and PM_{10} from the Taft NCore site (39-061-0040). It should be noted that although annual average ozone concentrations were used to define background levels for dry deposition calculations, health impact assessments were based on annual averages of the daily maximum 8-hour ozone concentrations, consistent with epidemiological concentration-response functions implemented in BENMAP-CE. Carbon dioxide dispersion was assumed negligible because climate change effects are based on global carbon levels, not spatially-specific concentrations.

Background ammonia (NH_3) concentrations are also required for the MESOPUFF II chemical transformation module and were specified using data from the National Atmospheric Deposition Program (NADP) Ammonia Monitoring Network (AMoN).[10] Monthly background ammonia concentrations used for the MESOPUFF II chemical mechanism were derived from the Oxford, Ohio Ammonia Monitoring Network site and specified as 0.478 ppb (January), 1.246 ppb (February), 1.329 ppb (March), 2.228 ppb (April), 2.655 ppb (May), 4.238 ppb (June), 2.506 ppb (July), 2.431 ppb (August), 2.990 ppb (September), 1.958 ppb (October), 1.719 ppb (November), and 0.986 ppb (December), calculated by duration-weighting bi-weekly passive-sampler observations by their temporal overlap with each month and converted assuming standard conditions (25C, 1 atm).

S2 Atmospheric Dispersion Modeling [adapted from Charles et al.[11]]

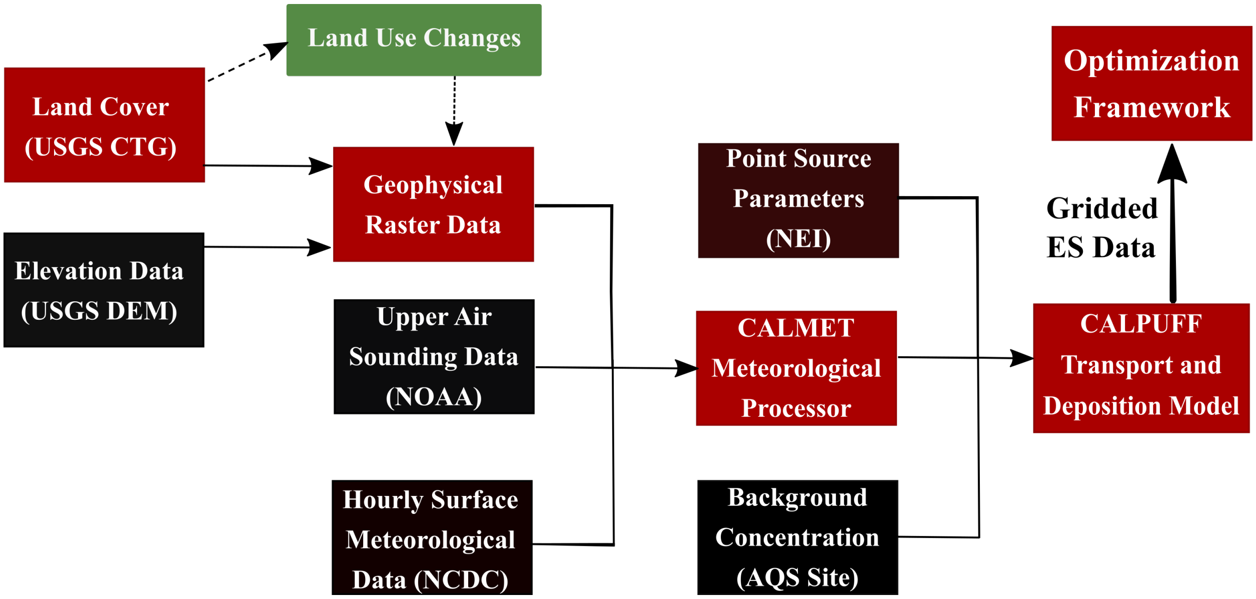

Figure S1: Atmospheric Dispersion Modeling System Process Flow and Data Input Sources. The boxes in black represent regional data that stay constant for a given facility. The green box shows the initial point of data manipulation for various land use scenarios while the red boxes represent all the steps affected by land use changes. USGS: United States Geological Survey, CTG: Composite Theme Grid (format), DEM: Digital Elevation Model, NOAA: National Oceanic and Atmospheric Administration, NCDC: National Climatic Data Center, NEI: National Emissions Inventory, AQS: Air Quality System, ES: Ecosystem Service. Reproduced with permission from Charles et al.[11] Copyright 2021 John Wiley and Sons.

The overall process of our application of the CALPUFF Modeling System can be found in Figure S1. This figure demonstrates the main data inputs and the simulation steps required to produce spatially-explicit ecosystem service, or dry deposition, data. Before simulating any chemical transport, a geophysical model is first created based on input land cover and elevation data. This data comes from the United States Geological Survey (USGS) Land Use and Land Cover Composite Theme Grid formatted data[12] and the USGS Digital Elevation Model[13]. As the geophysical model is created, a grid of receptors is created, resembling each spatial point at which the calculations will be conducted. The spacing between each receptor is equivalent to the spatial resolution at which land use changes can be considered. This grid is created simultaneously as the land cover and elevation data are processed with information specific to each receptor.

Similarly, the meteorological data at both the surface and upper air sounding levels are processed to be used in CALMET, the meteorological processor, along with the geophysical data. The upper air sounding data can be found from the National Oceanic and Atmospheric Administration / Earth Systems Research Laboratories Radiosonde Database[7] and the surface data can be found from the National Climatic Data Center Integrated Surface Hourly Data Base[6]. The upper air sounding data captures meteorological variables such as pressure and temperature at multiple heights from the surface, along a z-axis in the model. After all the geophysical and meteorological data are processed through CALMET, the point source data, dry deposition parameters, and background concentration data are added to the model for dispersion simulation. The point source data and background concentration are listed in Section S1 and the dry deposition parameters for NO_x are as follows: a diffusivity of 0.1656 cm^2/s, an alpha star of 1.0, a reactivity of 8.0, a mesophyll resistance of 5.0 s/cm, and Henry's Law coefficient of 3.5. The dry deposition parameters for

ozone are as follows: a diffusivity of 0.1440 cm^2/s, an alpha star of 1.0, a reactivity of 10.0, a mesophyll resistance of 0.0 s/cm, and Henry's Law coefficient of 0.01. These are the input requirements for simulating gaseous deposition in CALPUFF. For particle deposition, we used a geometric mass mean diameter of 0.48 µm and a standard deviation of 2.0 µm. These values are CALPUFF defaults.[9] After the CALPUFF model is applied, the results include both concentration and dry deposition calculations at each receptor defined in the model. Atmospheric chemistry was represented using the MESOPUFF II module embedded within CALPUFF, which simulates first-order gas-phase transformations, secondary particulate formation, and dry deposition of sulfur, nitrogen, and ozone species using prescribed background concentrations and meteorological conditions.

Because we are interested in simulating land use change (LUC) scenarios, Figure S1 also shows (in green) where the land cover data are manipulated from the existing land cover according to proposed scenarios, which will be introduced later. The boxes in red show all of the consecutive processes that must be executed again after the land cover data are manipulated for a given scenario, while the black boxes show all the data that remains the same for every land change scenario within the spatial boundaries of the simulation. Essentially, the red boxes show the downstream impacts of the modeling system due to changing the land cover data.

The full CALPUFF Modeling System available can include additional inputs that are omitted from our application such as: precipitation data for wet deposition, coastal impacts, over-water data, and others. Additional information on CALPUFF and the model system can be found in the User Guides, found at the following website: `https://www.epa.gov/scram/air-quality-dispersion-modeling-alternative-models`.

S3 Ecological Growth Dynamics

The ecological dynamics were simulated using the USFS Forest Vegetation Simulator (FVS), where growth of tree species most common to Ohio were simulated from sparse saplings to forest stand over 80 years. The simulated species were sugar maple, yellow-poplar, and white ash and the results are shown in Figure S2.

S4 Statistical Distribution of the Mortality Valuation

The EPA Standard Valuation Functions for 2021 are used to characterize the value of a statistical life (VSL) and available from the library integrated in the BENMAP-CE software. The mean value is reported at \$8.7 million and is characterized by a Weibull distribution. This distribution has the following probability density function[14]:

$$\left(\frac{\beta}{\alpha}\right)\left(\frac{x}{\alpha}\right)^{\beta-1} e^{-\left(\frac{x}{\alpha}\right)^{\beta}} \tag{1}$$

where α and β are parameters that characterize the uncertainty distribution. Based on 26 value-of-life studies that were analyzed in the 2021 EPA Standard Value Functions, we used an α value of 9,648,168 and a β value of 1.509588 with a mean value of \$8,705,114 (2015 USD), which assumes no cessation lag.[14]

S5 Optimization Formulation

Because our initial development of the TES Industrial Landscape Design Framework did not consider economic valuations of ecosystem services, we must add a term to subtract the benefits from the sum of the private costs, as follows

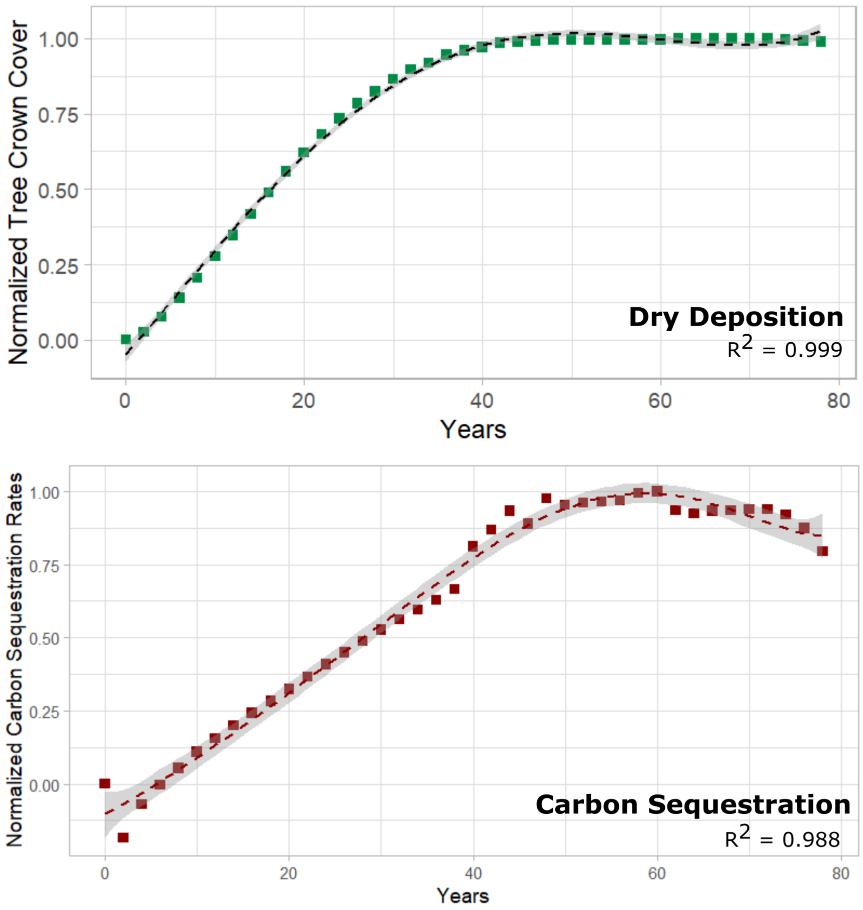

Figure S2: Normalized Tree Crown Growth and Carbon Sequestration Dynamics of Sugar Maple, Yellow-Poplar, and White Ash species. The black dashed line shows the curve of the fifth order polynomial regression equation and the red dots show the resulting data points from the US Forest Service Forest Vegetation Simulator.

$$\min_{S_{n,j},\, E_{n,i}} \sum_{n}^{N}\sum_{j}^{J} Z_{n,j} + \sum_{n}^{N}\sum_{i}^{I} Z_{n,i} - \sum_{n}^{N}\sum_{i}^{I} \hat{Z}_{n,i} \tag{2}$$

where S is the technological scaling variable, E is the ecological spatial decision variable, Z represents cost, n is the time step index, j is the technological option index, i is the receptor index, and $\hat{Z}$ represents the social benefits [$] that are attributed to the ecological, land-based solutions. $Z_{n,j}$ represents the total cost of technological solutions, $Z_{n,i}$ represents the cost of all the invested land areas, and $\hat{Z}_{n,i}$ represents the total valuated social benefits. This objective function both minimizes private costs and maximizes social benefits.

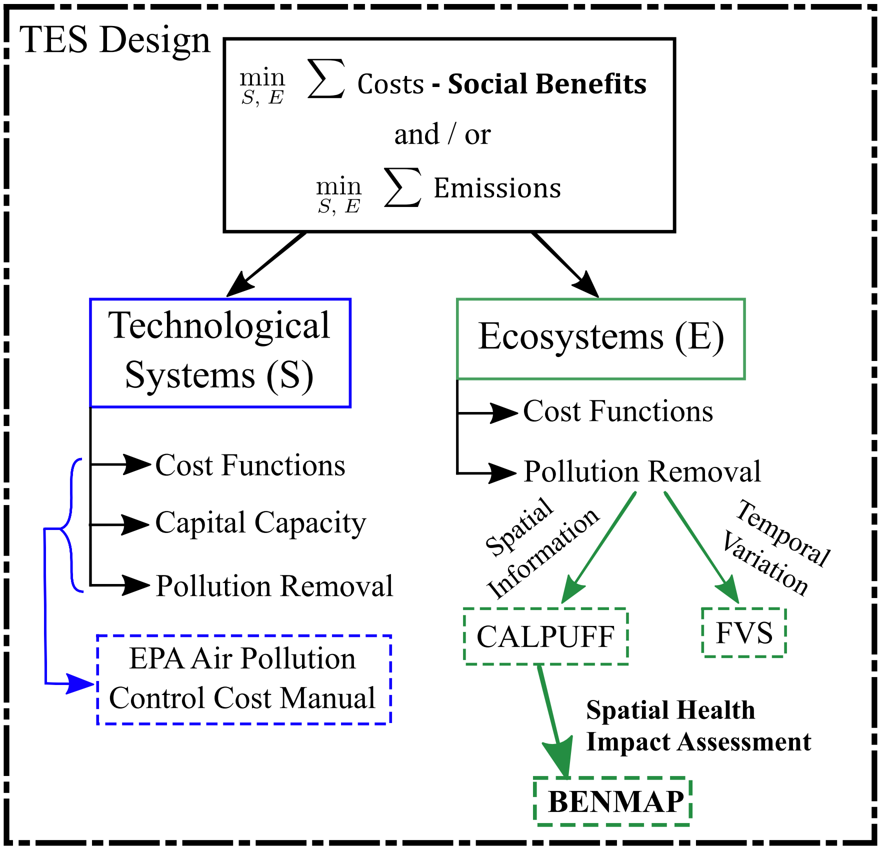

Figure S3: Overview of the TES industrial landscape framework with the additions of the social benefit evaluation and spatial health impact assessment, the added novelties of this work (shown in bold font). Adapted with permission from Charles et al.[11] Copyright 2021 John Wiley and Sons.

A simplified mathematical representation of the optimization program is as follows:

$$
\begin{aligned}
\min_{S_{n,j},\, E_{n,i}} \quad & \sum_{n}^{N}\sum_{j}^{J} Z_{n,j} + \sum_{n}^{N}\sum_{i}^{I} Z_{n,i} - \sum_{n}^{N}\sum_{i}^{I} \hat{Z}_{n,i} \\
\text{s.t.} \quad & \sum_{n}^{N} E_{n,i} \leq 1 \qquad \forall \quad i \in I \\
& K_{n,j} = \sum_{n}^{N} S_{n,\hat{j}} \qquad \forall \quad n \in T_{\hat{j}} \\
& S_{n,j^*} \leq K_{n,j} \\
& \hat{\chi}_{n,p} + \chi^*_{n,p} \geq Q^*_p \\
& Z, \hat{Z}, \hat{\chi}, \chi^* \equiv f(S_{n,j}, E_{n,j})
\end{aligned}
\tag{3}
$$

where S is the technological scaling variable, E is the ecological spatial decision variable, Z represents cost, n is the time step index, j is the technological option index, i is the receptor index, $\hat{Z}$ represents the social benefits [$] that are attributed to the nature-based solutions, K represents technological equipment capacity, T represents technological equipment life span, $\hat{j}$ represents capital investments, j^* represents technological operation, χ represents pollution removal, p is a chemical pollutant index, and Q represents

the pollution removal target. Further, $Z_{n,j}$ represents the total cost of technological solutions, $Z_{n,i}$ represents the cost of all the invested land areas, and $\hat{Z}_{n,i}$ represents the total valuated social benefits. $\hat{\chi}$ represents the technological removal of a given pollutant, p, and χ^* represents the ecological uptake of the pollutant. The first constraint ensures that across the ecological matrix, a given location can only be restored once across the time span of the simulation. The second constraint states that the operational capacity is a sum of capital investments for a given technology, j, within the limits of the life span of the equipment while the third constraint states that all technology must operate within the equipment capacity. The fourth constraint is that the sum of technological and ecological pollution uptake must equal the given target for pollutant p.

S6 Models of Air Pollution Control Units [from SI of Charles et al.[11]]

Selective Catalytic Reactor

Coal combustion processes most commonly rely on selective catalytic and selective non-catalytic reactors for post-combustion NO_x removal. The difference between the two is selective catalytic reactors rely on a catalyst to increase reactivity and enable operation at lower temperatures.[5]

Linearization. We apply a Taylor Series approximation to linearize the non-linear functions of SCR capital costs. After simplification of the equations presented in the Air Pollution Control Manual[3], the capital cost non-linear equation is as follows:

$$C_{n,S\hat{C}R} = 815,730\ x^{0.73} + 116.4\ x \tag{4}$$

where x is the power output of the facility in MW. The Taylor Series is then applied by setting x equal to 1,444 MW, the capacity of the power station. This results in the following linear equation.

$$C_{n,S\hat{C}R} = 83,636\ x + 44,605,600 \tag{5}$$

The error of the Taylor Series approximation was evaluated in our previous work[11], where yielding percent error was less than 1% within the operating limits of the SCR in the design results. Due to previous results, we assume the approximation error to be negligible.

To represent the fixed capital cost of the SCR unit, the following integer programming method was used:

$$\begin{aligned} \text{s.t. } & C_{n,S\hat{C}R} \geq 0 \\ & y_{S\hat{C}R} \in [0,1]^n \\ & \eta_{NO_x} = 9.8 \ \left[\frac{tonnes}{MW}\right] \\ & S_{n,S\hat{C}R} \leq 24,000\ [tonnes]\ y_{n,S\hat{C}R} \\ & C_{n,S\hat{C}R} = 83,636 \left[\frac{\$}{MW}\right]\ (S_{n,S\hat{C}R}/\eta_{NO_x})\ [MW] + 44,605,600\ [\$]\ y_{n,S\hat{C}R} \end{aligned} \tag{6}$$

where $y_{S\hat{C}R}$ introduces the integer programming variable at each step, n, so that the cost equals zero when S equals zero based on the presented constraints and the optimization's objective to minimize costs. In this form, $(S_{n,S\hat{C}R}/\eta_{NO_x})$ represents $x(t)$ in the Equation 4, as $S_{n,S\hat{C}R}$ describes the amount of NO_x removed in tonnes and η_{NO_x} is the conversion of tonnes of NO_x per MW of power output.

The operating cost for the selective catalytic reactor is much simpler as it is a linear function, as follows:

$$C_{n,SCR^*} = 6,027\ (S_{n,SCR^*}/\eta_{NO_x}) \tag{7}$$

where $(S_{n,SCR^*}/\eta_{NO_x})$ again represents power capacity.

Baghouse Filter

To remove particulate air pollution from flue gas, baghouse filters are commonly used in industry. These devices are fabric filters which can operate as either batch or semicontinuous processes with required offline time for cleaning.[4] The derived model for the baghouse filter technology from the EPA Air Pollution Control Cost Manual results in linear functions for both capital and operating costs. Similar, to the other technologies, these costs are a function scaled by power capacity [MW] of the generating station. Therefore, $(S_{n,B\hat{H}F}/\eta_{PM_{2.5}})$ is again used as a conversion from power to tonnes of pollution removed. This conversion is defined as

$$\eta_{PM_{2.5}} = 3.0 \left[\frac{tonnes}{MW}\right] \tag{8}$$

for $PM_{2.5}$ in units tonnes per MW. The capital cost equation is then defined as follows:

$$C_{n,B\hat{H}F} = 3,517\ (S_{n,B\hat{H}F}/\eta_{PM_{2.5}}) \tag{9}$$

with costs resulting in units of US \$. The operating cost is then calculated using the following equation.

$$C_{n,BHF^*} = 1,343\ (S_{n,BHF^*}/\eta_{PM_{2.5}}) \tag{10}$$

Carbon Capture Unit

The carbon capture unit was a simple linear model that assumed a cost of $29 per tonne of carbon capture. This value comes from an Intergovernmental Panel on Climate Change (IPCC) Report in 2018 that gave a range of 23-35 US$ for a carbon capture unit installed on a new integrated coal gasificiation combined cycle power.[15] This value was sized for 400-800 MW power plants (the largest in the report) and also assumed that unit was installed without retrofitting. This means that this value likely underestimates the cost for adding a CCS unit to an existing plant. For these case studies, the facility's goal is carbon neutrality so the minimum removal rate of carbon between technological and ecological systems is constrained to be greater than or equal to the emissions rate.

REFERENCES

[1] United States Envrionmental Protection Agency. Emissions & Generation Resource Integrate Database (eGRID), 2018. Available at: `https://www.epa.gov/egrid/download-data`. (Accessed on July 2021).

[2] United States Environmental Protection Agency. 2020 National Emissions Inventory (NEI) Flat Files, 2020. Available at: `https://www.epa.gov/air-emissions-inventories/2020-national-emissions-inventory-nei-data`. (Accessed Jan. 2026).

[3] Mussatti Daniel, S Ravi, MH Paula, et al. EPA Air Pollution Control Cost Manual. Technical report, United States Environmental Protection Agency Office of Air Quality Planning & Standards, 2002.

[4] Lawrence K Wang, Clint Williford, and Wei-Yin Chen. Fabric filtration. In *Air Pollution Control Engineering*, pages 59–95. Springer, 2004.

[5] Deepak Pudasainee, Vinoj Kurian, and Rajender Gupta. 2 - coal: Past, present, and future sustainable use. In Trevor M. Letcher, editor, *Future Energy (Third Edition)*, pages 21–48. Elsevier, third edition edition, 2020.

[6] National Climatic Data Center and National Oceanic and Atmospheric Administration. Standard Hourly Surface Data, 2024. Available at: `https://www.ncei.noaa.gov/data/global-hourly/`. (Accessed on December 2024).

[7] Earth's Research Systems Laboratory, National Oceanic and Atmospheric Administration, and Global Systems Division. Global Radiosonde Database, 2024. Available at: https://www.ncei.noaa.gov/data/integrated-global-radiosonde-archive/access/data-por/. (Accessed on December 2024).

[8] U.S. Environmental Protection Agency. Air Quality System (AQS) Data Mart: Daily Air Quality Data. https://www.epa.gov/outdoor-air-quality-data/download-daily-data, 2026. Accessed January 5, 2026.

[9] Joseph S Scire, David G Strimaitis, and Robert J Yamartino. A User's Guide for the CALPUFF Dispersion Model (Version 5). *Earth Tech. Inc*, (January):521, 2000.

[10] National Atmospheric Deposition Program (NADP). Ammonia Monitoring Network (AMoN). https://nadp.slh.wisc.edu/networks/ammonia-monitoring-network/, 2026. Accessed January 5, 2026.

[11] Michael Charles and Bhavik R Bakshi. Designing industrial landscapes for mitigating air pollution with spatially-explicit techno-ecological synergy. *AIChE Journal*, 67(10):e17347, 2021.

[12] United States Geological Survey. USGS CTG Land Use/Land Cover (LULC) Data. Available at: http://edcftp.cr.usgs.gov. (Accessed on October 2019).

[13] United States Geological Survey. USGS Digital Elevation Model (1/3 Arc Second). Available at: https://viewer.nationalmap.gov/basic/. (Accessed on October 2019).

[14] United States Environmental Protection Agency. BENMAP: Environmental Benefits Mapping and Analysis Program – Community Edition User Manual (Updated for BenMAP-CE Version 1.5.8). Technical report, United States Environmental Protection Agency Office of Air Quality Planning & Standards, 2023.

[15] Bert Metz, Ogunlade Davidson, HC De Coninck, Manuela Loos, and Leo Meyer. *IPCC special report on carbon dioxide capture and storage*. Cambridge: Cambridge University Press, 2005.
